# Mobilizing medical students for COVID-19 responses: Experience of Vietnam

**DOI:** 10.7189/jogh.10.020319

**Published:** 2020-12

**Authors:** Bach Xuan Tran, Long Hoang Vo, Hai Thanh Phan, Hai Quang Pham, Giang Thu Vu, Huong Thi Le, Carl A Latkin, Cyrus SH Ho, Roger CM Ho

**Affiliations:** 1 Institute for Preventive Medicine and Public Health, Hanoi Medical University, Hanoi, Vietnam; 2 Bloomberg School of Public Health, Johns Hopkins University, Baltimore, USA; 3 Institute for Global Health Innovations, Duy Tan University, Da Nang, Vietnam; 4 Faculty of Medicine, Duy Tan University, Da Nang, Vietnam; 5 Center of Excellence in Evidence-based Medicine, Nguyen Tat Thanh University, Ho Chi Minh City, Vietnam; 6 Department of Psychological Medicine, National University Hospital, Singapore, Singapore; 7 Department of Psychological Medicine, Yong Loo Lin School of Medicine, National University of Singapore, Singapore, Singapore; 8 Institute for Health Innovation and Technology (iHealthtech), National University of Singapore, Singapore, Singapore

To date, Coronavirus disease 2019 (COVID-19) pandemic has affected 185 countries and territories worldwide [1]. Community transmission has been recorded in a large number of countries and regions [2], which has led to a huge demand for human resources. Currently, countries’ health workforce has been in sharp focus, especially those are in preventive medicine, public health, and in treating and providing health care for infected people. Meanwhile, there has been great concern among researchers, organizations, and governments about the health care system’s ability to cope with COVID-19 [3]. According to International Labor Organization, there was a shortage in the number of workers in the health sector, especially in lower-middle income countries and low-income countries, resulting in disruption of health services [4,5]. The shortage of health workforce, particularly in developing countries, may hinder the delivery as well as management of health care services across the continuum of care during the pandemic, presenting a great challenge in COVID-19 control, both in hospitals and in the community.

Involvement of medical students in emergencies were reported in previous pandemics. In 1918, when the Spanish flu occurred, medical students filled in as nurses and interns, supplanting a large number of health professionals who were serving the troops [6]. Another example to be mentioned is the Copenhagen polio epidemic. In 1952, a Danish anesthetist demonstrated that patients with respiratory failure resulted from polio could survive if manually ventilated and thus, a total of 1400 medical students were recruited to ventilate patients constantly during the epidemic [7]. Given the experiences of mobilizing the “clinicians-in-training” from the past, many countries have incorporated medical students into the COVID-19 task force for more prompt responses to the pandemic. Medical schools in the United States, Italy, and the United Kingdom allow students to graduate early on the condition that they serve as frontline clinicians, while universities in Denmark keep medical students in their clinical placements and initiate fast-track courses in ventilator therapy and nursing assistance [8-10].

Vietnam preventive medicine has demonstrated certain advantages in preventing and controlling the COVID-19 outbreak. On the other hand, despite the wide coverage from central to grassroots levels, contact tracing and epidemiological investigations in Vietnam have revealed its weaknesses in terms of capacity and staff when many thousands of people are at risk of being infected. During the first week of April, Hanoi has rapidly traced over 26 000 people with a history of close contacts to confirmed COVID-19 cases at an early stage. Due to the complicated COVID-19 outbreak situation, medical universities in Vietnam have developed plans in case health staff or students are infected and proactively respond to possible scenarios. Based on a request of the Hanoi Department of Health, after opening training courses on essential medical understanding on COVID-19, Hanoi Medical University mobilized 97 senior students in preventive medicine and 27 final-year students of public health to support prevention, early detection, and control of COVID-19 with the health staffs at the Hanoi Center for Disease Control (CDC). Volunteer students at medical universities have been involved in the epidemiological investigation of cases coming from epidemic areas. They are providing counseling to people over the phone, collecting samples from suspected community groups, importing data into computers, and cleaning and disinfection for congregate settings for COVID-19.

The involvement of medical students in the prevention and control of COVID-19 has certain advantages. First and foremost, with a solid background on health care cultivated throughout years of in-depth education, senior medical students have sufficient practical and clinical capabilities. A variety of transferable skills, such as critical appraisal, observation, listening, logical reasoning, and decision making, gained through their practical training programs at hospitals as well as at outside health facilities, is also of great help. Furthermore, medical students have also been trained for effective interpersonal communication, understanding and treating serious illness, and ethical issues, which is helpful in investigating the epidemiological characteristics and symptomology. Finally, as medical students have been familiar with working under pressure for a long time, they are able to handle a heavy workload and perform well under high pressure. However, due to the differences between hospital and community environment, especially in such a pandemic as COVID-19, it might take the students quite a long time to become familiar with the work as well as the changing environment.

**Figure:**
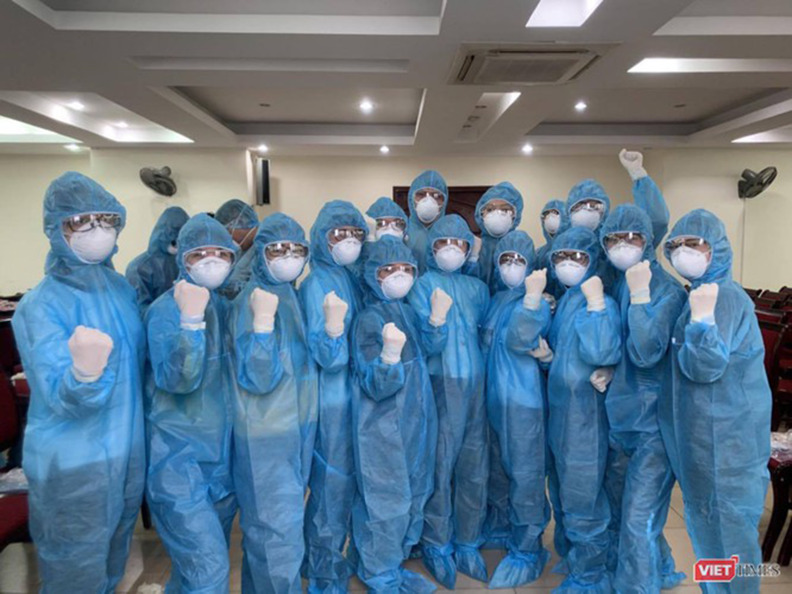
Photo: From Hanoi Medical University (used with permission).

The successful contact tracing model in Vietnam, using limited resources, suggests the importance of ensuring skillful human resources for pandemic preparedness. To prepare for medical students, universities should improve their training curriculums by incorporating field epidemiological practicum and engaging health authorities as supervisors throughout the programs. There should also be policies and protocols to be developed in the national emergency plan for pandemic responses that specifies medical students’ roles and responsibilities and coordinating mechanisms between universities and public health authorities. Since the pandemic is ongoing, the approach used in Vietnam might be helpful for other resource-scarce settings in conducting active and prompt responses in the pandemic.
